# Supplementary material for: Autoantibody Profiling for Accurate Differentiation of Type 1 and Type 2 Diabetes Mellitus in Omani Patients: A Retrospective Study
Source: Diagnostics (Basel). 2025 Sep 10;15(18):2296. doi: 10.3390/diagnostics15182296 (PMC12468106; doi:10.3390/diagnostics15182296)
Supplement: Supplementary file 1 [file diagnostics-15-02296-s001.zip › diagnostics-3773725-supplementary.pdf]

Supplementary Materials

This file contains supplementary figures and tables referenced in the main article titled: "Autoantibody Profiling for Accurate Differentiation of Type 1 and Type 2 Diabetes Mellitus in Omani Patients: A Retrospective Study."

Contents:

Figure S1. Mean Autoantibody Positivity Count Across Sex and Age Groups in T1DM and T2DM.
Figure S2. Mean Autoantibody Positivity by Diabetes Treatment Type.
Figure S3. Mean Autoantibody Positivity by Diabetes-Associated Complications
Table S1. Clinical and Immunological Characteristics of T1DM and T2DM Patients.

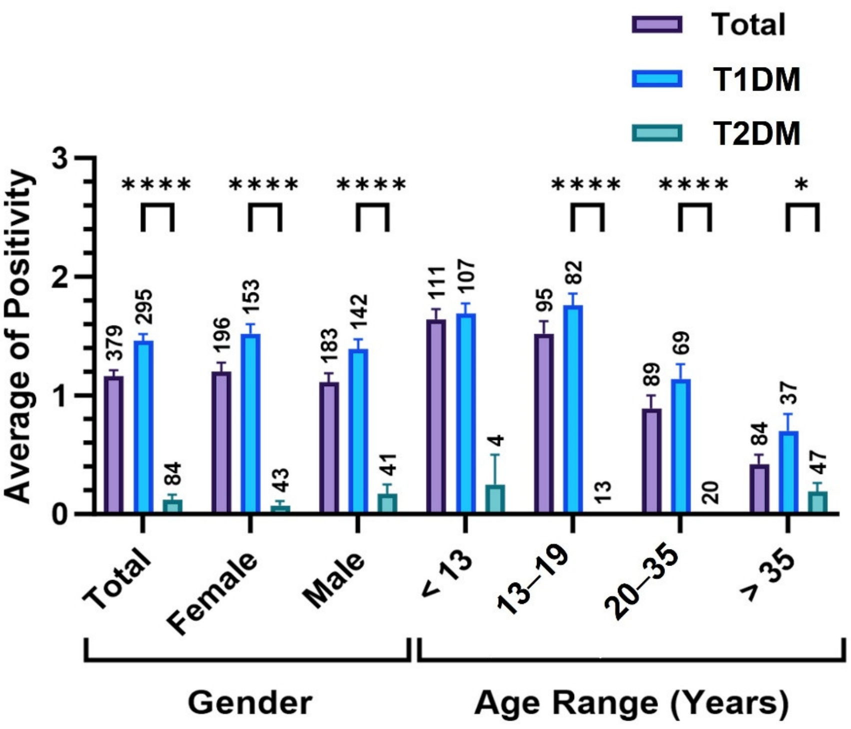

Figure S1. Mean Autoantibody Positivity Count Across Sex and Age Groups in T1DM and T2DM. Bar plots show the mean number of positive autoantibodies per patient stratified by sex and age groups (<13, 13–19, 20–35, >35 years) in the confirmed diabetes group comprising only verified T1DM or T2DM cases (Total), and by diabetes subtype (T1DM, T2DM). Statistical significance is indicated by asterisks (*$p < 0.05$; **$p < 0.01$; ***$p < 0.001$; ***$p <$ *0.0001).* Error bars represent the SEM, and sample sizes are indicated above each bar.

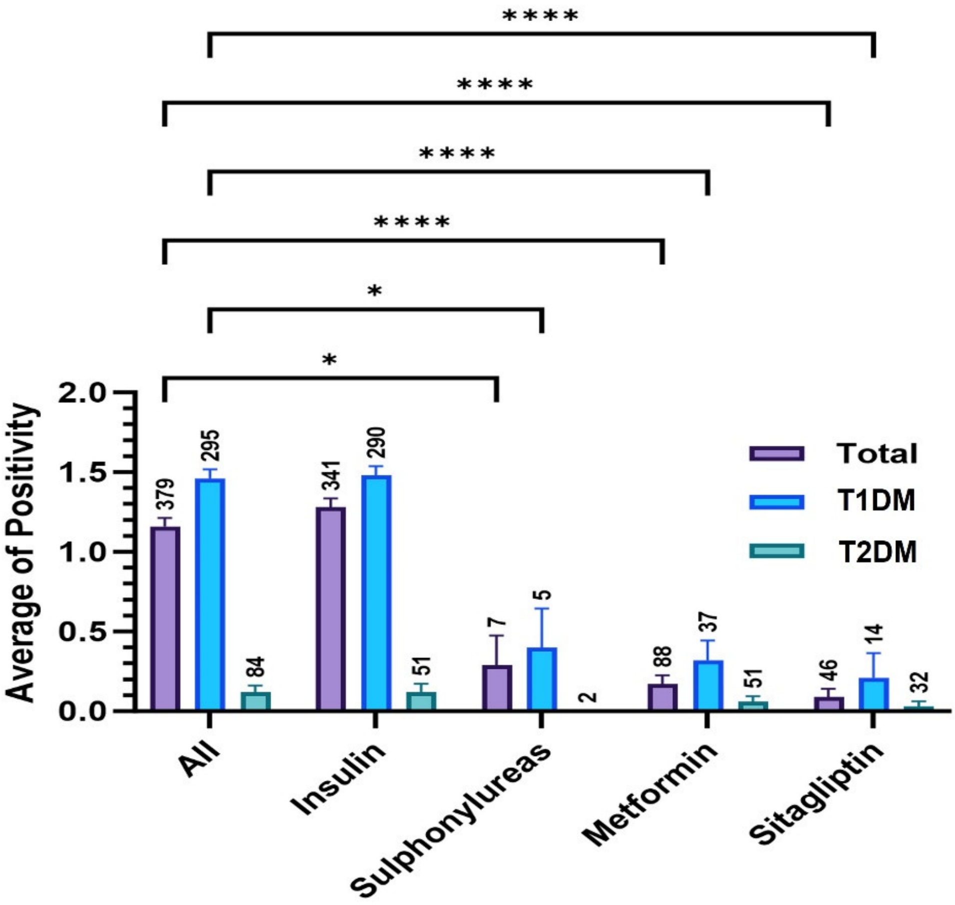

Figure S2. Mean Autoantibody Positivity by Diabetes Treatment Type. Bar plots illustrate the mean number of positive autoantibodies per patient across different treatment modalities (insulin, sulfonylureas, metformin, and sitagliptin). Data are presented for the entire cohort (All), the confirmed diabetes group comprising only verified T1DM or T2DM cases (Total), and by diabetes subtype (T1DM and T2DM). Statistical significance between treatment groups is indicated by asterisks (*$p < 0.05$; ****$p < 0.0001$). Error bars denote the SEM, and the number of patients in each category is shown above each bar.

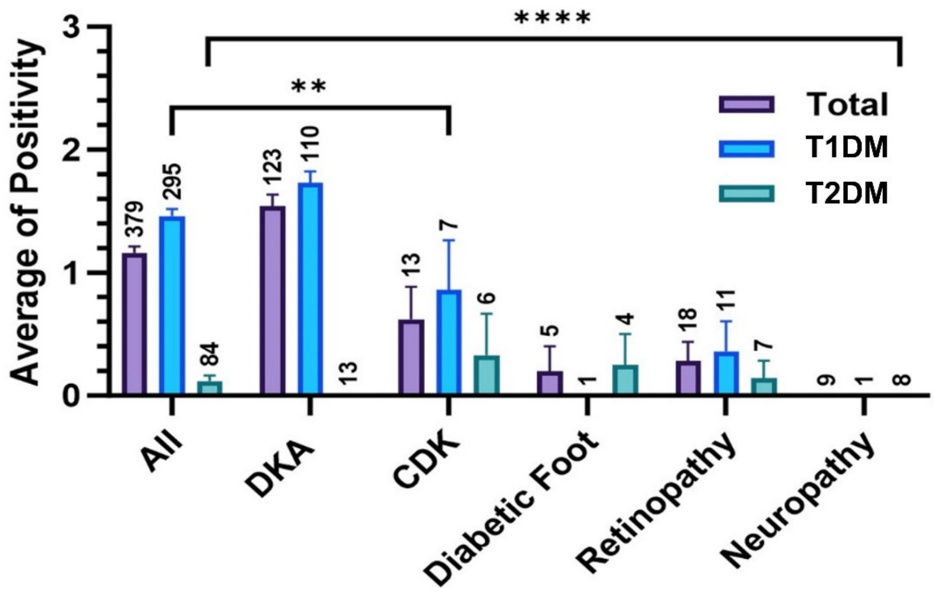

Figure S3. Mean Autoantibody Positivity by Diabetes-Associated Complications. Bar plots illustrate the mean number of positive autoantibodies per patient across various diabetes-related complications (DKA, CKD, retinopathy, neuropathy, and diabetic foot). Data are presented for the entire cohort (All), the confirmed diabetes group comprising only verified T1DM or T2DM cases (Total), and by diabetes subtype (T1DM and T2DM). Statistical significance between subgroups is indicated by asterisks (**$p < 0.01$; ****$p < 0.0001$). Error bars represent the SEM, and sample sizes are shown above each bar.

Table S1. Clinical and Immunological Characteristics of T1DM and T2DM Patients. Summary of key demographic, clinical, and immunological features in patients with T1DM or T2DM. Parameters include age (years, mean ± SD, *n*), sex distribution (%, *n*), HbA1c (%, mean ± SD, *n*), frequency of HbA1c ⩾ 8.0% (%, *n*), and autoantibody positivity rates (anti-Islet, anti-GAD, anti-TPO, anti-tissue; %, *n*). The mean number of positive autoantibodies per patient is also shown. P-values were obtained using t-tests (continuous) or chi-square tests (categorical), as appropriate. *n*. refers to the number of patients who have been tested.

Marker	T1DM	T2DM	p value
Age (mean ± SD, *n*.)	19 ± 13, 299	36±16, 84	< 0.001
Female (%, *n*.)	78.4, 156	21.6, 43	-
Male (%, *n*.)	77.7, 143	22.3, 41	-
HbA1c (mean ± SD, *n*.)	10.45 ± 2.37, 297	9.26 ± 2.36, 83	< 0.001
HbA1c (%, *n*.)	83 ,253	17, 52	< 0.001
Anti-islet (%, *n*.)	98.4, 184	1.6, 3	< 0.001
Anti-GAD (%, *n*.)	97.1, 203	2.9, 6	< 0.001
Anti-TPO (%, *n*.)	97.2, 35	2.8, 1	-
Anti-tissue (%, *n*.)	100, 17	0, 0	-
Positivity (mean ± SD, *n*.)	1.47 ± 0.99, 299	0.12 ± 0.39, 84	< 0.001
